# Genetic and Pharmacological Factors That Influence Reproductive Aging in Nematodes

**DOI:** 10.1371/journal.pgen.0030025

**Published:** 2007-02-16

**Authors:** Stacie E Hughes, Kimberley Evason, Chengjie Xiong, Kerry Kornfeld

**Affiliations:** 1 Department of Molecular Biology and Pharmacology, Washington University School of Medicine, St. Louis, Missouri, United States of America; 2 Division of Biostatistics, Washington University School of Medicine, St. Louis, Missouri, United States of America; Stanford University, United States of America

## Abstract

Age-related degenerative changes in the reproductive system are an important aspect of aging, because reproductive success is the major determinant of evolutionary fitness. Caenorhabditis elegans is a prominent organism for studies of somatic aging, since many factors that extend adult lifespan have been identified. However, mechanisms that control reproductive aging in nematodes or other animals are not well characterized. To use C. elegans to measure reproductive aging, we analyzed mated hermaphrodites that do not become sperm depleted and monitored the duration and level of progeny production. Mated hermaphrodites display a decline of progeny production that culminates in reproductive cessation before the end of the lifespan, demonstrating that hermaphrodites undergo reproductive aging. To identify factors that influence reproductive aging, we analyzed genetic, environmental, and pharmacological factors that extend lifespan. Dietary restriction and reduced insulin/insulin-like growth factor signaling delayed reproductive aging, indicating that nutritional status and a signaling pathway that responds to environmental stress influence reproductive aging. Cold temperature delayed reproductive aging. The anticonvulsant medicine ethosuximide, which affects neural activity, delayed reproductive aging, indicating that neural activity can influence reproductive aging. Some of these factors decrease early progeny production, but there is no consistent relationship between early progeny production and reproductive aging in strains with an extended lifespan. To directly examine the effects of early progeny production on reproductive aging, we used sperm availability to modulate the level of early reproduction. Early progeny production neither accelerated nor delayed reproductive aging, indicating that reproductive aging is not controlled by use-dependent mechanisms. The implications of these findings for evolutionary theories of aging are discussed.

## Author Summary

In animals, aging is characterized by degenerative changes that progressively diminish the function of tissues and organs. Degenerative changes in life support systems eventually cause death, whereas degenerative changes in reproductive systems eventually cause the cessation of progeny production. Successful reproduction is the ultimate purpose of animal life, and therefore it is important to determine the causes of reproductive aging and the way reproductive aging has been sculpted by natural selection during evolution. Because most aging studies focus on somatic degeneration and lifespan, relatively little is known about the causes of reproductive aging. To identify and characterize factors that influence reproductive aging, we used the nematode worm *C. elegans,* which is a prominent model for studies of somatic aging. Our results indicate that reproductive aging in worms can be delayed by cold temperature, by restricting nutrient uptake, by diminishing insulin signaling, and by an anticonvulsant medicine that acts on the nervous system. These studies identify genetic pathways and environmental factors that influence reproductive aging. Surprisingly, reproductive aging was not influenced by progeny production early in the reproductive period, indicating that using the germ line to produce progeny does not accelerate degenerative changes. These results suggest that reproductive aging is not caused by use-dependent mechanisms.

## Introduction

Aging is characterized by widespread degenerative changes in tissue morphology and function that result in a progressive diminishment of the ability to reproduce and survive. An experimental approach that has led to important insights into causes of these degenerative changes is the identification of factors that influence lifespan of model organisms such as yeast, nematodes, and flies. For example, the lifespan of the free-living soil nematode C. elegans can be significantly extended by cold temperature [1], by mutations that affect mitochondrial function and insulin/insulin-like growth factor (IGF) signaling [2–4], and by drugs such as anticonvulsant medicines [5]. Analyses of how these factors influence lifespan have clarified the causes of age-related declines of systems that are necessary for life support. Because lifespan is the focus of many aging studies, age-related declines of systems that do not contribute to life support have been characterized less extensively. The analysis of factors that delay aging of systems that do not contribute to life support can elucidate the causes of aging for these systems and define relationships between the degenerative changes that occur in different systems.

The age-related decline of reproductive function, which we refer to as reproductive senescence or reproductive aging, is important for several reasons. Reproductive aging is likely to play a critical role in evolution. The reproductive history of an individual involves (1) the initiation of progeny production as a result of sexual maturity, (2) some level of progeny production during the fertile period, and (3) the cessation of progeny production as a result of reproductive aging or death. These three processes determine the rate of progeny production and the total number of progeny generated by an individual. Because successful reproduction is the ultimate purpose of animal life, it is likely that all three processes are sculpted by natural selection during evolution. Thus, understanding reproductive aging is likely to provide critical insights into the evolution of aging.

If reproductive aging were delayed, then an animal would be predicted to generate more progeny. Because aging in general and reproductive aging in particular appear to diminish progeny production, these traits have been a subject of debate for evolutionary theorists. Some of the first notable theories proposed that it was advantageous for the group if older, “worn out” individuals died, as exemplified by the writings of Weismann [6]. These theories have been questioned because they rely on group selection, not individual selection [7]. A major criticism is that “cheaters,” mutants that display delayed aging and thereby generate more progeny, will have a selective advantage. The modern theory of the evolution of aging was proposed by Medawar and postulates that extrinsic mortality is the cause of the evolution of aging, because it results in an age-related decline in the force of natural selection [8]. This theory proposes that natural selection cannot favor traits that extend longevity beyond the time when most individuals have died as a result of extrinsic mortality. Williams' elaboration of this basic idea, the antagonistic pleiotropy theory, postulates that traits that promote early reproduction at the cost of diminishing late reproduction will be favored by natural selection [9]. This theory proposes that tradeoffs exist between early and late reproduction, and selection for enhanced early reproduction is the cause of aging. An assumption of the theories of Medawar and Williams is that aging confers a selective disadvantage because it decreases progeny production. Medawar wrote concerning a mutation that causes degeneration, “If differences in its age of onset are indeed genetically determined, then natural selection must so act as to postpone it, for those in whom the age of onset is relatively late will, on the average, have had a larger number of children than those afflicted by it relatively early, and so will have propagated more widely whatever hereditary factors are responsible for the delay” [8]. Williams wrote, “I shall assume initially, therefore, that senescence is an unfavorable character, and that its development is opposed by selection” [9]. The assumption that continued progeny production confers a selective advantage, and therefore reproductive aging confers a selective disadvantage, is a critical aspect of these theories. An alternative assumption is that progeny production is advantageous initially but at some point continued progeny production confers a selective disadvantage; based on this assumption, reproductive aging confers a selective advantage because it halts progeny production. This assumption would be the basis for different theories for the evolution of aging.

Medawar and Williams did not perform experimental tests of these theories, but they described testable predictions of these theories. A key prediction of the Medawar theory is that high levels of extrinsic mortality will correlate with a shorter lifespan, whereas low levels of extrinsic mortality will correlate with a longer lifespan. Reznick and colleagues explored this prediction by analyzing guppies from Trinidad [10]. Populations of guppies that live in downstream areas of rivers experience high rates of extrinsic mortality, since a predatory fish is present. By contrast, populations of guppies that live upstream are isolated from this predator and experience low rates of extrinsic mortality. Guppies from these populations were cultured in the lab to determine lifespan and reproductive characteristics. Guppies that evolved in conditions of high extrinsic mortality displayed an extended life span compared to guppies that evolved in conditions of low extrinsic mortality [10]. This is a surprising finding, since it does not appear to be consistent with the prediction of the theory of Medawar. In addition to an extended lifespan, guppies that evolved in conditions of high extrinsic mortality displayed an earlier onset of reproduction, a higher rate of progeny production, and delayed reproductive aging, resulting in the generation of significantly more progeny [10]. Thus, guppies exposed to high predation evolve delayed reproductive aging, an adaptation that enables greater progeny production. These findings raise the possibility that in a habitat with high predation, natural selection favors guppies that generate a larger number of progeny, which may compensate for progeny lost to predation. One mechanism to increase progeny production is to delay reproductive aging, and guppies with delayed reproductive aging may be favored by natural selection in the habitat with high predation. By contrast, in a habitat with low predation, natural selection may favor guppies that generate a smaller number of progeny, since fewer progeny are killed by predation. In this habitat guppies with an earlier onset of reproductive aging may have a selective advantage.

A key prediction of the antagonistic pleiotropy theory is that genetic variants with increased late reproduction should not display normal levels of early reproduction but rather should display decreased early reproduction. Williams wrote, “Successful selection for increased longevity should result in decreased vigor in youth” [9]. Because this prediction of the theory can be tested experimentally, it has become important to analyze the relationships between early and late progeny production. One approach to testing this prediction has been selecting genetic variants of *Drosophila* with increased late reproduction. These experiments have not always yielded the same results, but in several cases strains selected for enhanced late reproduction displayed reduced early reproduction [11–13]. While the identification of strains with reduced early progeny production and enhanced late progeny production is consistent with the theory, it does not demonstrate that the theory is correct, since these findings may also be consistent with other theories. By contrast, the identification of strains with normal early progeny production and enhanced late progeny production would be inconsistent with the theory of antagonistic pleiotropy.

In addition to its role in evolution, progeny production and reproductive aging have important relationships to somatic aging. One relationship that has been examined extensively is the impact of progeny production on somatic aging [14]. It has been proposed that resources allocated for progeny production are not available for somatic maintenance, and progeny production thereby accelerates somatic aging. Alternatively, progeny production may inflict somatic damage, thereby shortening lifespan. Consistent with these theories, dietary restriction can reduce progeny production and extend the lifespan of C. elegans self-fertile hermaphrodites [1], *Drosophila* [15], and rodents [16–18]. Furthermore, some single gene mutations that extend lifespan also reduce progeny production in C. elegans self-fertile hermaphrodites [2,3], *Drosophila* [19], and rodents [20]. In C. elegans, ablation of the germ cells extends lifespan if the somatic gonad is intact, but not if the somatic gonad is also ablated [21]. In a group of human females, a small number of progeny positively correlates with extended longevity [22]. However, the correlation between reduced progeny number and extended lifespan is not always observed. Among wild-type *Drosophila,* there is generally a positive correlation between progeny production and longevity [23]. Furthermore, there are single gene mutations that extend *Drosophila* or self-fertile hermaphrodite *C. elegans* lifespan but do not reduce progeny production [24–27]. It is possible that these mutations reduce progeny production in specific culture conditions that have yet to be determined [28,29]. A second relationship is the impact of somatic aging on reproductive function. Reproductive aging may occur primarily because of degenerative change in the germ line. However, reproduction is an integrated function that requires the soma, and reproduction may decline, at least in part, because of declines in somatic function. In rodents, ovary transplantation experiments indicate that age-related changes in estrous cycling arise from both ovarian and somatic impairments [30,31]. A third relationship is the extent to which common mechanisms influence the processes of degenerative change in somatic and reproductive tissues. In principle, the same factors might influence somatic aging and reproductive aging, or separate factors might control these events.

Studies of reproductive aging have been conducted in a variety of animals. In humans, females display an age-related decrease in the number of oocytes in the ovary [32]. Human females typically experience menopause, the complete cessation of progeny production, in the fifth decade of life. Female rodents also display an age-related cessation of cycling and progeny production. In rodents, dietary restriction inhibits reproductive cycling, but interestingly when full feeding is resumed in mid-life, the cessation of reproductive cycling and progeny production is delayed [16–18]. In fruit flies, several factors have been demonstrated to influence reproductive aging. Reproductive aging can be delayed by dietary restriction [33, 34] and some single gene mutations [35,36]. In males, reproductive activity can accelerate reproductive aging [37].

Studies of reproductive aging of C. elegans have focused primarily on self-fertile hermaphrodites. Wild-type hermaphrodites first produce approximately 300 self-sperm and then produce eggs [38]. Self-fertile hermaphrodites efficiently use these self-sperm and deposit about 250 self-fertilized progeny. When the supply of self-sperm is exhausted, hermaphrodites deposit unfertilized oocytes and then they cease to deposit oocytes [38]. Hermaphrodites that are mated to males use male sperm in preference to self-sperm and can produce a larger number of progeny, indicating that the number of self-fertile progeny that can be generated is limited by the number of self-sperm that are produced [39].

Reproductive aging in self-fertile hermaphrodites has been measured using two basic approaches. The first is analyzing age-related changes in the morphology of the germ line. Garigan et al. [40] used Nomarski optics to demonstrate that older, self-fertile hermaphrodites display more widely spaced nuclei in the mitotic germ line and nucleoplasm that is disrupted by cavities and grainy material. Older gonads frequently appeared to be shriveled, containing relatively few nuclei and cellularized nuclei. These changes began to be apparent at the fifth day of adulthood and increased with age. An insulin/IGF-signaling pathway regulates life span of C. elegans and several other animals [41]. Loss-of-function mutations in genes encoding DAF-2, a receptor tyrosine kinase, and AGE-1, a component of the PI-3 kinase, increase life span [2,3]. A major target of this pathway is the forkhead transcription factor DAF-16, and a *daf-16(lf)* mutation decreases life span and suppresses the life span extension caused by *age-1* and *daf-2* mutations [3]. Garigan et al. [40] showed that age-related changes in germ cell morphology in self-fertile hermaphrodites were accelerated in *daf-16(lf)* mutants and delayed in *daf-2(lf)* mutants.

The second approach to measuring reproductive aging is analyzing the schedule of progeny production. The typical summary statistics include the duration of the progeny production period and the number of progeny generated at the end of the reproductive period. Several studies have examined how mutations that affect insulin/IGF signaling influence progeny production of self-fertile hermaphrodites. Some *daf-2* mutations cause sterility of self-fertile hermaphrodites, indicating that *daf-2* is necessary for reproductive development. The site of action of *daf-2* was investigated by genetic mosaic analysis, and *daf-2* is required cell nonautonomously to control reproductive development [42]. *daf-2* activity influences the time of initiation of progeny production; reducing *daf-2* function with RNA interference in self-fertile hermaphrodites delays the onset of progeny production, and time of administration studies suggest that *daf-2* may function late in development to affect the onset of reproduction [43]. The effects of insulin/IGF signaling on reproductive aging have been analyzed in self-fertile hermaphrodites. Huang et al. [44] reported that the self-fertile reproductive period of *daf-2*, *age-1,* and *daf-16* mutants is not significantly different from wild-type hermaphrodites. Larsen et al. [24] showed that for self-fertile *daf-2* hermaphrodites with extended life spans, reproduction is neither delayed nor prolonged, except for the observation that *daf-2(e1370)* mutants intermittently produced a small number of progeny late in life. Gems et al. [26] showed that for self-fertile hermaphrodites, late progeny were produced by multiple strains with class 2 *daf-2* alleles, but not by long-lived strains with class 1 *daf-2* alleles. Late progeny production in self-fertile *daf-2* hermaphrodites was also reported by Tissenbaum and Ruvkun [25]. The period of self-fertile reproduction is extended by a mutation of *tph-1* that reduces serotonin production and likely acts upstream of *daf-16* [45]. Late progeny production or an extended reproductive span in a mutant self-fertile hermaphrodite compared to a wild-type self-fertile hermaphrodite demonstrates that the mutation delays sperm depletion, since self-sperm depletion causes reproductive cessation in wild-type self-fertile hermaphrodites. The mutation might also delay the age-related decline of reproductive capacity. However, to demonstrate such a delay, it is important that both the experimental and control hermaphrodites have a sufficient supply of sperm. Factors that can delay reproductive aging of C. elegans hermaphrodites that are not sperm limited have not been reported.

Here, we demonstrate that reproductive aging occurs in mated C. elegans hermaphrodites that are not sperm limited, establishing the utility of this model system for studies of reproductive aging. We identified four factors that can delay reproductive aging of mated hermaphrodites raised at 20 °C with abundant food: cold temperature, dietary restriction, a mutation that reduces the activity of the insulin/IGF-signaling pathway, and an anticonvulsant medicine that likely affects neural activity. By manipulating sperm availability, we demonstrated that early progeny production neither accelerated nor delayed the age-related decline of progeny production. These studies identify novel factors that control reproductive aging and have implications for the evolution of reproductive aging.

## Results

### Measurements of Reproductive Aging in Mated C. elegans Hermaphrodites

**Figure 1:**
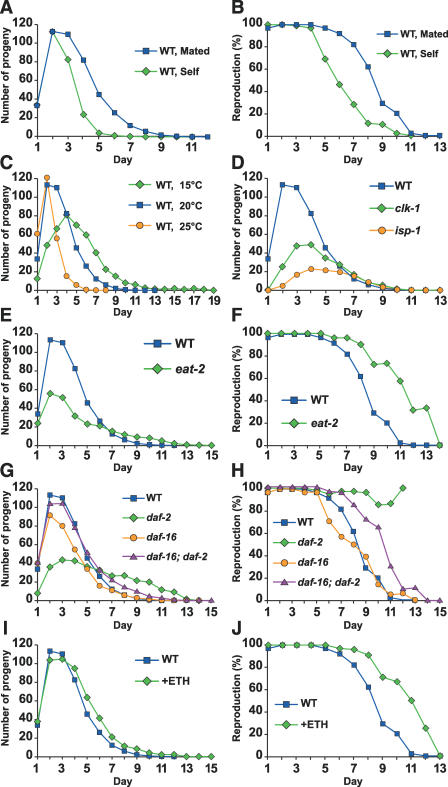
Reproductive Activity of C. elegans Hermaphrodites (A), (C–E), (G), and (I) show average daily progeny production of live hermaphrodites. (B), (F), (H), and (J) show the percentage of live hermaphrodites producing progeny. The *daf-2* plot (H) does not end at zero percent because no *daf-2* hermaphrodites survived beyond day 12. Number of hermaphrodites at the start of the experiment is presented in Table 1. Hermaphrodites were mated for days 1 and 2 to three wild-type (WT) males, except data labeled Self. Studies were conducted at 20 °C, except data labeled 15 °C and 25 °C. The mutant alleles were *isp-1(qm150*)*, clk-1(qm30*)*, eat-2(ad465), daf-2(e1370*)*,* and *daf-16(mu86*)*.* Wild-type hermaphrodites were exposed to 2 mg/ml ethosuximide (+ETH) from conception until death (I and J).

To measure reproduction, we typically placed a single fourth larval stage (L4) hermaphrodite on a petri dish with abundant food, transferred the animal to a fresh dish daily, and scored the number of live progeny produced daily. Wild-type hermaphrodites cultured at 20 °C begin to deposit fertilized eggs about 12 h after the L4 stage. This method provides accurate and precise measurements of daily progeny production. An examination of these data revealed that there is a vertex or peak of progeny production that separates a period of increasing reproductive function from a period of declining reproductive function (Figure 1). We used the L4 stage, the vertex, and the cessation of progeny production to define periods of reproductive function: the reproductive growth span is the phase with an increasing rate of progeny production and is defined as the time from L4 until the vertex, and the total reproductive span is defined as the time from L4 to the cessation of progeny production. The increasing progeny production that occurs during the reproductive growth span indicates that there is a developmental program that enhances reproductive function and culminates in a peak of progeny production. The declining progeny production that occurs next indicates that the reproductive system undergoes age-related degeneration.

**Table 1:**
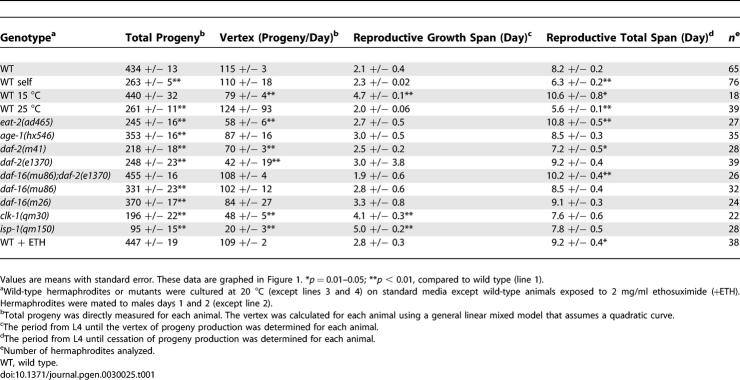
Progeny Production and Reproductive Spans of Early Mated Hermaphrodites

**Figure 2:**
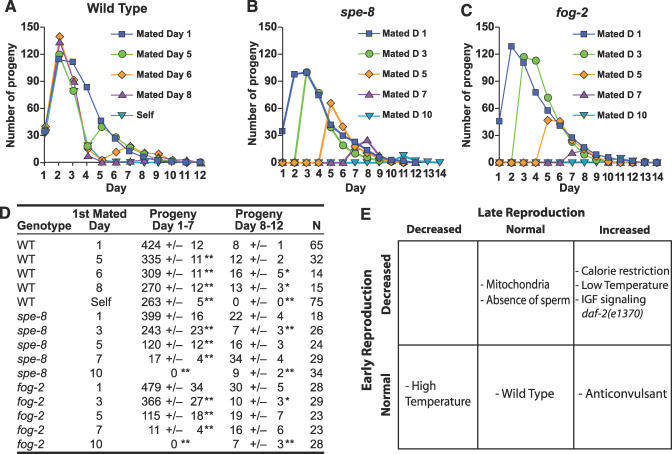
Reproductive Aging Is Independent of Early Progeny Production Wild-type (A), *spe-8(hc50)* (B), or *fog-2(q71)* (C) hermaphrodites were mated to wild-type (WT) males for 24–48 h starting with the day specified. Graphs show daily progeny production, and (D) shows the average total progeny production (+/−standard error) for days 1–7 and days 8–12. N, the number of animals examined. * and ** represent a *p*-value of 0.01−0.05 and <0.01, respectively, compared to the mated day 1 value of the same genotype. (E) A summary of the effects on early and late reproduction of an anticonvulsant drug, temperature, and mutations that affect mitochondrial function, caloric intake, and IGF signaling.

These data were analyzed to yield quantitative measurements of early progeny production and late progeny production for each individual. Because the vertex of progeny production typically occurs early in the total reproductive span, this value is a useful measure of early progeny production. To quantitatively measure early reproduction, we estimated the time of the vertex and the level of progeny production at the vertex (see Materials and Methods; Table 1). To quantitatively measure reproductive aging, we used two approaches. First, the total reproductive span was used to determine the duration of the reproductive period. Second, the number of progeny generated at the end of the reproductive period was determined. (Figure 2; Table 2). If a factor delays reproductive aging, then it is expected to extend the total reproductive span and increase the number of progeny generated at the end of the reproductive period.

**Table 2:**
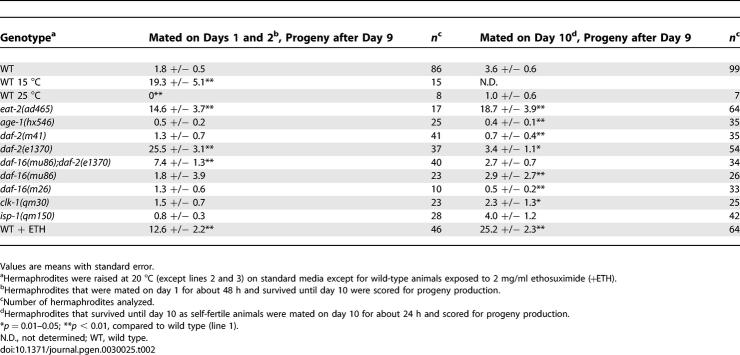
Quantification of Late Reproduction

Hermaphrodites produce approximately 300 self-sperm at the start of gametogenesis before switching to oocyte production [38,46]. The number of progeny generated by self-fertile hermaphrodites appears to be limited by the number of self-sperm. First, the number of sperm corresponds closely to the number of self-progeny generated, indicting that nearly every sperm is utilized to produce a zygote [38]. By contrast, hermaphrodites produce oocytes in excess, and unfertilized oocytes begin to be laid as sperm is depleted. Second, in hermaphrodites that are mated to males, the male sperm is used in preference to the self-sperm, and mated hermaphrodites can produce substantially more than 300 progeny [39]. These observations demonstrate that hermaphrodites have reproductive capacity that is not utilized when they are limited to approximately 300 self-sperm but can be utilized when they are provided sufficient male sperm.

We reasoned that for live progeny production to be an accurate measure of reproductive capacity at the end of the reproductive period, it is critical that hermaphrodites have abundant sperm continually, so that they do not cease live progeny production as a result of sperm limitation. To develop a method for providing sufficient sperm to hermaphrodites, we cultured hermaphrodites with males for different intervals during the reproductive period. Hermaphrodites cultured continually with males displayed a shortened lifespan and frequently died during the reproductive period (unpublished data), consistent with the findings of Gems and Riddle [47]. These observations suggest that continual exposure to males results in traumatic injury to hermaphrodites, probably as a result of repeated mating, and this trauma can lead to premature death. To reduce traumatic injury, we determined the minimum period of time that a hermaphrodite could be exposed to males and still receive sufficient male sperm for the duration of the lifespan. Hermaphrodites were mated to males for different periods of time, and the sex ratio of progeny at the end of the reproductive period was monitored. Self-fertile hermaphrodites generate almost all hermaphrodite progeny, whereas mated hermaphrodites generate half male and half hermaphrodite progeny. Hermaphrodites mated to males for 24–48 h beginning at the L4 stage frequently produced both male and hermaphrodite progeny until the cessation of reproduction, indicating that these hermaphrodites never exhaust the supply of male sperm. Occasionally, mated hermaphrodites produced male progeny briefly and then produced only hermaphrodite progeny. These hermaphrodites probably received a small supply of male sperm that was used initially, and then they reverted to the use of self-sperm. To insure that the hermaphrodites that were included in the data analysis had an abundant supply of male sperm, we monitored the sex ratio of the progeny and only analyzed mated hermaphrodites that continued to produce male progeny until reproductive cessation.

Hermaphrodites mated on days 1 and 2 might acquire sufficient sperm initially, but the sperm might become inviable over time, leading to a decrease in progeny production. To address this issue, we compared hermaphrodites mated on day 1 to hermaphrodites mated late in life. If male sperm becomes inviable over time, then hermaphrodites mated day 1 might produce fewer progeny late in life than hermaphrodites that are mated late in life and receive a supply of fresh sperm. By contrast, if male sperm remains viable for the duration of the reproductive period, then hermaphrodites mated day 1 and hermaphrodites mated late are predicted to generate similar numbers of progeny late in life. Figure 2D shows that hermaphrodites mated on days 3, 5, 6, 7, or 8 usually had a similar number of progeny late in life compared to hermaphrodites mated on day 1; specifically, in five comparisons the values were not significantly different, in two comparisons the late mated value was slightly higher, and in two other comparisons the late mated value was slightly lower. These observations indicate that male sperm received by a hermaphrodite on day 1 does not become inviable during the reproductive period.

Self-fertile, wild-type hermaphrodites cultured at 20 °C had an average of 263 total progeny (the range was 87–354) (Figure 1A and 1B; Table 1). These values are consistent with efficient utilization of approximately 300 self-sperm [38,46]. The vertex of progeny production was 110 progeny/day and occurred at 2.3 d. The total reproductive span was 6.3 d. Compared to self-fertile hermaphrodites, wild-type hermaphrodites mated on days 1 and 2 had a greater total progeny number of 434 (the range was 139–690), a 65% increase (Figure 1A and 1B; Table 1). The vertex of progeny production was similar, 115 progeny/day at 2.1 d. The total reproductive span was increased to 8.2 d, a 30% increase. These findings indicate that early progeny production is similar in self-fertile and mated hermaphrodites, whereas late progeny production is increased in mated hermaphrodites. These results suggest two important conclusions. First, measuring progeny production underestimates the reproductive capacity of self-fertile hermaphrodites at the end of the reproductive period; hermaphrodites must be mated to avoid sperm depletion and to use progeny production as an accurate measure of reproductive aging. Second, age-related declines of progeny production occur in mated hermaphrodites that have sufficient sperm. Therefore, reproductive aging occurs in mated C. elegans hermaphrodites and is caused by factors other than sperm depletion.

### Genetic, Environmental, and Pharmacological Factors That Influence Reproductive Aging

To characterize mechanisms that influence reproductive aging, we analyzed manipulations that affect life span including mutations, a drug, and temperature. These treatments can be classified by their effects on early reproduction (normal or decreased) and late reproduction (normal, decreased, or increased) (Figure 2E). Three treatments decreased early reproduction and increased late reproduction. One treatment was cold temperature. The life span of poikilotherm animals like C. elegans is increased by cold temperature [1]. The reproductive span of self-fertile hermaphrodites is extended by cold temperature, indicating that this treatment delays sperm depletion [1,44]. Hermaphrodites mated early and cultured at 15 °C took longer to reach the vertex of progeny production and displayed a reduced vertex of 79 progeny/day, demonstrating that culture at 15 °C reduces early progeny production (Figure 1C; Table 1). The total number of progeny was 440 (range of 95–626), similar to hermaphrodites cultured at 20 °C. Hermaphrodites cultured at 15 °C had an increased total reproductive span of 10.6 d, a 29% increase. These hermaphrodites produced 19.3 progeny after day 9 compared to 1.8 progeny after day 9 for hermaphrodites raised at 20 °C, an 11-fold increase (Table 2). The extended total reproductive span and the increased progeny production late in life indicate that cold temperature delays reproductive aging.

A second treatment was dietary restriction. Dietary restriction extends the life span of many animals, including C. elegans [1]. The *eat-2(ad465)* loss-of-function mutation reduces pharyngeal pumping rate and food intake and extends adult life span about 47% [48,49]. This *eat-2* mutation extends the reproductive span of self-fertile hermaphrodites, indicating that dietary restriction delays sperm depletion [44]. *eat-2(ad465)* mated hermaphrodites displayed a reduced vertex of 58 progeny/ day, demonstrating a decrease in early progeny production (Figure 1E and 1F; Table 1). The total number of progeny was significantly reduced to 245 (the range was 106–416). The total reproductive span increased to 10.8 d for mated *eat-2* hermaphrodites, a 32% increase. Progeny production after day 9 was increased 8-fold and 5-fold in early and late mated *eat-2* hermaphrodites, respectively (Table 2). These findings indicate that dietary restriction delays reproductive aging.

A third treatment was reducing activity of an insulin/IGF-signaling pathway. This pathway regulates life span of C. elegans and several other animals [41]. *daf-2(e1370)* is a partial loss-of function mutation that affects the kinase domain of the DAF-2 receptor tyrosine kinase, and this mutation causes a dramatic extension of adult lifespan [3]. Mated *daf-2(e1370*) hermaphrodites had a reduced vertex of 42 progeny/day, demonstrating a decrease in early progeny production (Figure 1G and 1H; Table 1). Total progeny production was reduced significantly to 248 (the range was 19–493). Nearly every *daf-2(e1370)* animal mated early in life died because of internal hatching of progeny; this occurs at a lower frequency in mated wild-type hermaphrodites and it complicates the interpretation of the total reproductive span. For progeny production after day 9, the *daf-2(e1370)* mutation caused a 14-fold increase for hermaphrodites that were mated early and survived until day 10, but caused no significant effect for hermaphrodites mated late (Table 2). Overall, these findings indicate that *daf-2(e1370*) delays reproductive aging. A *daf-16* mutation partially suppressed the affects of *daf-2* on late reproduction (Figure 1G and 1H; Table 2). Two additional mutations that extend life span to a lesser degree, *daf-2(m41)* and *age-1(hx546),* did not significantly delay reproductive aging (Tables 1 and 2). Thus, reducing the activity of the insulin/IGF-signaling pathway can delay reproductive aging, but the effect was only caused by a specific mutation.

One class of mutations decreased early reproduction but did not significantly affect reproductive aging. Loss-of-function mutations in genes important for mitochondrial function such as *clk-1(qm30)* and *isp-1(qm150)* extend adult life span about 20% and 63%, respectively [4,50]. *clk-1* hermaphrodites display an extended self-fertile reproductive span, indicating that there is a delay in sperm depletion [44]. Mated *clk-1* and *isp-1* hermaphrodites took longer to reach the vertex of progeny production and had a reduced vertex, indicating that early progeny production is decreased compared to wild type (Figure 1D; Table 1). The number of total progeny was reduced significantly for *clk-1* to 196 (range of 41–374) and for *isp-1* to 95 (range of 4–326), consistent with previous reports that the total number of progeny generated by self-fertile hermaphrodites is reduced [51]. *clk-1* and *isp-1* hermaphrodites did not have an extended total reproductive span and did not produce significantly more progeny than wild type after day 9 (Tables 1 and 2), indicating that these mutations do not delay reproductive aging. Thus, reduced early progeny production in a long-lived mutant is not sufficient to delay reproductive aging.

One treatment resulted in normal early reproduction and decreased late reproduction, culture at the elevated temperature of 25 °C. Early mated hermaphrodites raised at 25 °C had a vertex of 124 progeny/day, similar to animals cultured at 20 °C (Figure 1C; Tables 1 and 2). The total number of progeny generated by hermaphrodites cultured at 25 °C was reduced significantly to 261 (the range was 111–466). The total reproductive span was decreased 32%, and fewer progeny were produced after day 9 in early-mated hermaphrodites, suggesting that culture at 25 °C accelerates reproductive aging.

One treatment resulted in normal early reproduction and increased late reproduction, the anticonvulsant ethosuximide. Exposure to 2 mg/ml ethosuximide extends adult life span about 17%, and ethosuximide is likely to function by affecting neuronal activity [5]. Ethosuximide treatment does not significantly affect the self-fertile reproductive span, indicating that drug treatment does not delay sperm depletion [5]. Mated hermaphrodites treated with ethosuximide had a vertex of 109 progeny/day, similar to untreated animals. These hermaphrodites produced a higher total progeny number of 447 (the range was 193–723), although this trend did not achieve statistical significance with this sample size (Figure 1I and 1J; Table 1). Ethosuximide treatment increased the total reproductive span to 9.2 d, a 12% increase. The number of progeny produced after day 9 increased by 7-fold in both early and late mated hermaphrodites (Table 2). These results demonstrate that ethosuximide treatment delays reproductive aging.

### Early Progeny Production Does Not Accelerate or Delay Reproductive Aging

To address the relationships between early progeny production and reproductive aging using an alternative approach, we used sperm availability to manipulate hermaphrodite reproduction. Wild-type hermaphrodites mated on day 1 generated 424 progeny during the early and middle reproductive period (days 1–7) and 8 progeny late in life (days 8–12) (Figure 2A and 2D). Hermaphrodites that were mated on progressively later days 5, 6, and 8 displayed significantly decreased progeny production days 1–7, but displayed only minor changes in late progeny production.

Hermaphrodites with the *spe-8(hc50)* mutation produce nonfunctional sperm and thus produce no viable self-progeny. *spe-8(hc50)* hermaphrodites do mature and ovulate unfertilized oocytes at the normal rate, thus incurring the metabolic costs of reproduction. s*pe-8(hc50)* hermaphrodites mated to wild-type males on day 1 produced 421 progeny, a number similar to mated wild-type hermaphrodites, and they displayed a pattern of reproductive aging similar to wild type (Figure 2B). *spe-8* hermaphrodites mated on progressively later days 3, 5, 7, or 10 displayed dramatically reduced progeny production days 1–7 (Figure 2B and 2D). Notably, the age-related decline in progeny production was similar for each mating day, and late progeny production was independent of the mating day.

Because wild-type and *spe-8* hermaphrodites still produce many fertilized or unfertilized oocytes early, we examined *fog-2(q71)* hermaphrodites that produce no self-sperm and very few unfertilized oocytes, since the oocyte maturation signal from sperm is lacking [52]. *fog-2* hermaphrodites ovulate at a much-reduced rate, and thus the metabolic effects of continual oocyte production are much less than wild type or *spe-8* mutants. *fog-2(q71*) hermaphrodites mated day 1 produced 509 progeny, a number similar to wild-type hermaphrodites. *fog-2(q71)* hermaphrodites mated on days 3, 5, 7, or 10 displayed dramatic reductions in early progeny production, but did not display increased late progeny production. Early and late mated hermaphrodites displayed similar patterns of age-related declines in progeny production (Figure 2C and 2D). These studies demonstrate that early reproduction does not accelerate or delay reproductive aging and indicate that the timing of reproductive aging is independent of the substantial metabolic demands of reproductive activity. In other words, the reproductive system undergoes an age-related decline in function that appears to be independent of whether the system is used to generate progeny.

## Discussion

Age-related changes of C. elegans reproduction have been analyzed primarily in self-fertile hermaphrodites. The first important issue addressed by our studies of mated hermaphrodites is whether C. elegans hermaphrodites undergo reproductive aging. Self-fertile hermaphrodites display a decline of progeny production beginning about day 2.3, and progeny production ceases about day 6.3. The cessation of progeny production occurs because the supply of self-sperm is completely utilized [38]. Sperm depletion might also contribute to the declining progeny production; alternatively, this decline might be caused by other factors. To address this issue, we compared hermaphrodites that are self-fertile to hermaphrodites that were mated early in life. Mated hermaphrodites displayed a decline in progeny production that begins about day 2.1, indicating that the time of initiation of the decline in progeny production is not influenced by sperm depletion. The decline in progeny production is more gradual in mated hermaphrodites than in self-fertile hermaphrodites, and the cessation of progeny production is delayed until day 8.2 in mated hermaphrodites. These results indicate that sperm depletion in self-fertile hermaphrodites accelerates the decline in progeny production and causes the cessation of progeny production. By contrast, the decline in progeny production displayed by mated hermaphrodites is not caused by sperm depletion. The mated hermaphrodites that we analyzed had sufficient sperm, since they continued to produce cross progeny until the time of reproductive cessation. Furthermore, the male sperm does not appear to become inviable over time, since mated hermaphrodites displayed this decline of progeny production even when they received a fresh supply of sperm late in life. These results demonstrate that the reproductive system in mated hermaphrodites undergoes an age-related decline in function independent of sperm availability.

Several prominent theories of aging are based on the concept that using an organ system promotes age-related degeneration. Potential mechanisms include the accumulation of mechanical damage and the accumulation of deleterious products of metabolism, such as reactive oxygen species [53]. For example, contracting the muscular pharynx of C. elegans has been proposed to contribute to age-related declines of pharyngeal function [54]. To investigate how using the reproductive system to produce oocytes affects age-related degeneration of reproductive function, we manipulated progeny production by controlling sperm availability. To obtain complete control over early progeny production, we utilized *fog-2* mutants that are self-sterile and behave like females; they produce no fertilized oocytes and few unfertilized oocytes in the absence of male sperm, so they do not incur the metabolic costs of progeny production until they are mated [52]. When male sperm is available, *fog-2* hermaphrodites produce about the same number of progeny as mated wild-type hermaphrodites, indicating that germ-line function is normal with the exception of self-sperm production. If progeny production causes the accumulation of damage or deleterious metabolites that contribute to reproductive aging, then *fog-2* hermaphrodites that produce no progeny early in life as a result of sperm limitation are predicted to have delayed reproductive aging. By contrast, if progeny production early in life does not cause reproductive aging, then *fog-2* hermaphrodites that produce no progeny early in life as a result of sperm limitation are predicted to have a normal time course of reproductive decline. Our results with *fog-2* and two other strains clearly demonstrate that early reproduction neither accelerates nor delays reproductive decline. This result is striking, because progeny production is a major metabolic activity for C. elegans hermaphrodites. These results indicate that reproductive aging in C. elegans hermaphrodites is not controlled by use-dependent mechanisms and may be controlled by time-dependent mechanisms. The relationships between early progeny production and reproductive aging have not been widely studied, but it has been examined in *Drosophila* males. High levels of early mating accelerate the decline of late reproduction, suggesting that use-dependent mechanisms influence reproductive aging in *Drosophila* males [37]. There may be sex or species specific differences in the relationships between early reproduction and reproductive aging.

Caloric restriction extends the lifespan of many animals and delays a wide range of age-related degenerative changes. We determined how caloric restriction affects reproductive aging of mated C. elegans hermaphrodites by analyzing *eat-2* mutants that have defective feeding, resulting in reduced caloric intake and an extended lifespan [48]. Self-fertile *eat-2* hermaphrodites display an extended reproductive span, indicating that there is delayed sperm depletion [44]. Consistent with this observation, caloric restriction caused by the *eat-2* mutation reduced early progeny production and the total number of progeny generated by mated hermaphrodites. Furthermore, mated *eat-2* mutant hermaphrodites displayed an extended reproductive period and increased progeny production late in life, indicating that caloric restriction delays reproductive aging. These effects of caloric restriction on C. elegans are similar to the effects of caloric restriction on reproduction of females of other species. The only intervention that has been well documented to extend the reproductive period of female rodents is caloric restriction [16–18]. Caloric restriction also reduces the total number of progeny produced by female rodents. Similarly, dietary restriction of female *Drosophila* reduces the number of progeny generated early and the total number of progeny, and in some cases late reproduction is increased [33,34]. While caloric restriction reduces the number of progeny produced early in life by C. elegans hermaphrodites and other animals, our results with sperm manipulation indicate that this does not cause the delayed reproductive aging of *C. elegans.* We conclude that the reduced progeny production early in life and the delayed reproductive aging are likely to be independent affects of caloric restriction. Caloric restriction delays somatic and reproductive aging in worms, rodents, and flies, indicating that caloric restriction may influence a conserved mechanism that influences both somatic and reproductive tissues.

Ambient temperature influences the lifespan of poikilotherm animals such as C. elegans [1]. Compared to the typical culture temperature of 20 °C, higher temperatures such as 25 °C shorten lifespan and lower temperatures such as 15 °C extend lifespan. Lower temperatures also extend the reproductive span of self-fertile hermaphrodites, indicating that sperm depletion is delayed [1,44]. Our analysis of mated hermaphrodites showed that hermaphrodites cultured at 15 °C displayed an extended period of reproduction and higher levels of reproduction late in life compared to hermaphrodites cultured at 20 °C. These results indicate that cold temperature delays reproductive aging. Cold temperature did not affect the total number of progeny significantly. Prominent theories that address the mechanisms of delayed aging caused by reduced temperature include (1) a global effect on the rate of chemical reactions and (2) the induction of a stress response. The result that temperature affects both reproductive and somatic aging is consistent with the model that these effects are mediated by similar mechanisms. Further studies are necessary to define these mechanisms.

An evolutionarily conserved insulin/IGF-signaling pathway has a major influence on the adult lifespan of C. elegans, and this pathway also influences the lifespan of other animals [41]. Mutations that affect this pathway have a variety of effects on reproduction of self-fertile hermaphrodites; specific alleles of *daf-2* can result in the production of self-progeny late in life [24–26] and delay age-related morphological changes in the gonad [40], indicating that reducing the activity of this pathway can delay reproductive aging. By contrast, several mutations that cause an extended lifespan do not extend the self-fertile reproductive span [24,44]. Our results show that reducing the activity of this pathway can delay reproductive aging in mated hermaphrodites. The effect was caused by the *daf-2(e1370)* mutation, but not by other mutations that cause lesser extensions of lifespan. The effects of this *daf-2* mutation were partially suppressed by a loss-of-function mutation of *daf-16*. A pathway involving *daf-2* and *daf-16* affects dauer formation and adult lifespan [3], and our results suggest that a similar pathway affects reproductive aging. Reducing the activity of this pathway also reduced the level of early progeny production and the total number of progeny. The reduced level of early progeny production and the delayed reproductive aging are probably independent effects, since reducing early progeny production by sperm limitation does not delay reproductive aging.

The anticonvulsant medicine ethosuximide extends C. elegans adult lifespan and delays age-related declines of somatic functions [5]. Ethosuximide is likely to influence somatic aging by modulating neural activity. Ethosuximide treatment does not extend the reproductive period of self-fertile hermaphrodites, indicating that sperm depletion is not delayed [5]. In mated hermaphrodites, ethosuximide did extend the period of reproduction and increased late progeny production, indicating that this drug delays reproductive aging. Drugs that delay reproductive aging of C. elegans or other animals have not been previously described. These results indicate that neural activity influences the aging of non-neuronal reproductive cells, suggesting that neural activity acts non-cell autonomously to control aging. Interestingly, ethosuximide treatment did not decrease early progeny production and increased the total number of progeny modestly. Other factors that increase total progeny production of mated C. elegans hermaphrodites have not been reported, but a similar phenomenon has been observed in other animals. Mutations in the *Drosophila* genes *Indy* and *EcR* that increase life span can increase the total number of progeny and number of progeny produced late in life [35,36]. Mating in the ant Cardiocondyla obscurior increases both early and late reproduction [55]. These studies indicate that C. elegans and several other animals have the capacity for additional late reproduction, which is not normally utilized.

The relationships between factors that influence reproductive and somatic aging have not been well characterized. Here we demonstrate that four factors that delay somatic aging also delay reproductive aging. These findings suggest that there is a substantial overlap between factors that control these processes. However, several mutations that extend C. elegans lifespan did not delay reproductive aging. One possible interpretation is that some processes affect somatic aging but not reproductive aging. Another possible interpretation is that these processes affect both somatic and reproductive aging, but the mutations we analyzed reveal the effect on somatic aging but not the effect on reproductive aging.

The results of these studies have implications for understanding the evolution of reproductive aging. First, our results address the relationships between early progeny production and reproductive aging and are relevant to predictions of the antagonistic pleiotropy theory. The antagonistic pleiotropy theory predicts that increased progeny production late in the reproductive period should be accompanied by decreased progeny production early in the reproductive period [9]. We identified factors that increase late reproduction and decrease early reproduction, consistent with this prediction of the theory. In addition, a factor was identified that increases late reproduction but does not decrease early reproduction, and this is not consistent with this prediction of the theory. Overall, these results suggest that there is no consistent relationship between early progeny production and late progeny production. Therefore, selection during evolution for high levels of early reproduction may not be a cause of reproductive aging in C. elegans.

**Figure 3:**
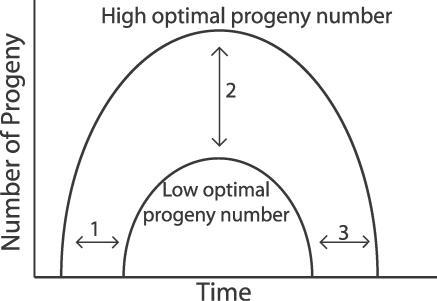
Reproductive Aging Contributes to the Ability of Animals to Generate the Optimal Progeny Number Hypothetical progeny production curves illustrate three mechanisms for controlling progeny number: the timing of the onset of progeny production (labeled 1), the level of steady-state progeny production (labeled 2), and the timing of cessation of progeny production or reproductive aging (labeled 3). Reproductive aging might be sculpted during evolution by selection for animals that generate the optimal number of progeny.

Our results indicate that there is plasticity in the timing of reproductive aging, and we speculate that selection for an optimal number of progeny may determine the timing of reproductive aging. First, C. elegans hermaphrodites that are not sperm limited undergo a complete cessation of progeny production as a result of reproductive aging before the end of the lifespan. A similar pattern is displayed by other female animals, such as humans and guppies [56]. These findings suggest that somatic aging may not limit reproduction, but rather the reproductive system is engineered to fail before the soma fails. In addition, our results indicate that C. elegans hermaphrodites have the capacity to generate a larger number of total progeny and a larger number of progeny later in life, and these capacities are not normally utilized. Studies of other animals reveal similar extra capacity [35,36,55]. Together, these findings indicate that C. elegans is not engineered to generate the maximum possible number of progeny. We speculate that there is an optimal number of F1 progeny, and reproductive aging contributes to the ability of the animal to generate the optimal progeny number (Figure 3). If a species encounters selective pressure for an increase in progeny number, then delayed reproductive aging may evolve. If a species encounters selective pressure for a decrease in progeny number, then accelerated reproductive aging may evolve. According to this theory, environmental factors that establish the optimal progeny number will be critical for the evolution of reproductive aging. Limiting progeny production to an optimal number might act at two levels to maximize reproductive success. At the level of the individual, generating the optimal progeny number may limit competition for resources among the progeny and maximize the probability that the optimal number of F1 progeny mature to become reproductive adults. At the level of the population, generating the optimal progeny number may help to achieve the maximum sustainable number of animals in the population and avoid oscillations in the number of animals in the population that might increase vulnerability to extinction. Further studies are necessary to determine how reproductive aging contributes to the ability of animals to generate the optimal number of progeny and influences population dynamics.

## Materials and Methods

### General methods and strains.

C. elegans were cultured on 6-cm petri dishes containing NGM agar and a lawn of Escherichia coli strain OP50 at 20 °C unless stated otherwise [57].

*daf-2(e1370 P1465S)* and *daf-2(m41 G383E)* are partial loss-of-function mutations that affect the kinase domain and ligand-binding domain of the DAF-2 receptor tyrosine kinase, respectively [58,59]; *age-1(hx546)* is a partial loss-of-function mutation in the AGE-1 phosphatidylinositol-3-OH (PI3) kinase [60]; *daf-16(m26)* is a partial loss-of-function mutation that disrupts mRNA splicing, and *daf-16(mu86)* is a strong loss-of-function mutation caused by a deletion in the DAF-16 forkhead transcription factor [61,62]; *eat-2(ad465)* is a strong loss-of-function or null mutation that causes a deletion of the first 45 amino acids of the EAT-2 non-alpha nicotinic acetylcholine receptor [49]; *isp-1(qm150 P225S)* is a loss-of-function mutation that affects the iron sulfur protein of mitochondrial complex III [50]; *clk-1(qm30)* is partial loss-of-function mutation that affects the homologue of yeast CAT5/Coq7 [63]; *fog-2(q71 Y148Stop)* is a possible null mutation in an F-box containing protein that binds to the GLD-1 protein during spermatogenesis [64]; *spe-8(hc50)* is a mutation that affects a nonreceptor protein-tyrosine kinase required for hermaphrodite spermatogenesis. We generated the *daf-16(mu86); daf-2(e1370)* strain using standard techniques.

### Analysis of fertility and reproductive aging.

Hermaphrodites were synchronized by selecting animals at the fourth larval stage based on the appearance of the vulva as a dark half circle under a dissecting microscope. L4 hermaphrodites were placed on individual petri dishes (time zero) and transferred to fresh dishes every day until death or at least four days without progeny production. Progeny were counted using a dissecting microscope about two days after transfer. For mating experiments, three young, wild-type males were added to the dish and removed after two days for days 1–5, or after one day for days 6–10 (unless stated otherwise). For matings on days 1–8, hermaphrodites that did not mate to males were recognized by a lack of male progeny and excluded from the data. Sterile hermaphrodites were also excluded from the data. Fertile animals that died during the experiment were included in the data until the day of death for Figure 1 and Table 1, and *n* values are the number of animals at the start of the experiments.

For experiments in Table 2, we mated L4 hermaphrodites to multiple males on days 1 and 2 either individually or in groups of two to six hermaphrodites. Hermaphrodites were transferred to fresh petri dishes every second day until day 10 and then placed on individual petri dishes for progeny scoring. For the matings on day 10, hermaphrodites were transferred individually or in groups of two to six every second day until day 10; on day 10 hermaphrodites were placed on individual plates with three males for one day and then transferred as necessary for progeny scoring. For both protocols, hermaphrodites that died before day 9 were excluded.

### Experiments using pharmacological compounds.

Ethosuximide was obtained from Sigma Chemical (http://www.sigmaaldrich.com) and added to molten nematode growth medium as described [5]. Parent hermaphrodites were exposed to the drug starting from the L4 stage, and self-progeny were maintained on drug-containing dishes for the duration of the experiments.

### Determination of vertex and spans and statistical analysis.

The estimation of the vertex on the longitudinally measured progeny is through a general linear mixed model [65], which assumes a quadratic growth curve for the progeny over time. The implementation of this model is by PROC MIXED/SAS [66]. The equality of time of vertex (reproductive growth span) and progeny level at vertex between wild type and any other group is tested using delta method based on the general linear mixed model (Table 1). In order to avoid the bias from the floor effect on the estimation of the vertex, we did not use small progeny observations at the end of the reproductive span in the general linear mixed model. The total progeny and the total reproductive span (Table 1), progeny after day 9 (Table 2), and progeny days 1–7 and days 8–12 (Figure 2) are computed for each worm and compared between wild type and any other group by the Wilcoxon's rank sum test.

## Abbreviations

IGF: insulin-like growth factor
L4: fourth larval stage
